# An update on advances in magnetic resonance imaging of multiple system atrophy

**DOI:** 10.1007/s00415-018-9121-3

**Published:** 2018-11-20

**Authors:** Viorica Chelban, Martina Bocchetta, Sara Hassanein, Nourelhoda A. Haridy, Henry Houlden, Jonathan D. Rohrer

**Affiliations:** 1 Department of Neuromuscular Diseases, UCL Institute of Neurology, Queen Square, London, WC1N 3BG UK; 2 Department of Neurology and Neurosurgery, Institute of Emergency Medicine, Toma Ciorbă 1, 2052 Chisinau, Moldova; 3 Dementia Research Centre, Department of Neurodegenerative Disease, UCL Institute of Neurology, Queen Square, WC1N 3BG London, UK; 4 Diagnostic Radiology department, Faculty of Medicine Assiut University, Assiut, Egypt; 5 Department of Neurology and Psychiatry, Faculty of Medicine, Assiut University Hospital, Assiut, Egypt; 6 Department of Brain, Repair and Rehabilitation, UCL Institute of Neurology, Queen Square, WC1N 3BG London, UK

**Keywords:** Multiple system atrophy, MRI, Imaging, Neurodegeneration

## Abstract

In this review, we describe how different neuroimaging tools have been used to identify novel MSA biomarkers, highlighting their advantages and limitations. First, we describe the main structural MRI changes frequently associated with MSA including the ‘hot cross-bun’ and ‘putaminal rim’ signs as well as putaminal, pontine, and middle cerebellar peduncle (MCP) atrophy. We discuss the sensitivity and specificity of different supra- and infratentorial changes in differentiating MSA from other disorders, highlighting those that can improve diagnostic accuracy, including the MCP width and MCP/superior cerebellar peduncle (SCP) ratio on T1-weighted imaging, raised putaminal diffusivity on diffusion-weighted imaging, and increased T2* signal in the putamen, striatum, and substantia nigra on susceptibility-weighted imaging. Second, we focus on recent advances in structural and functional MRI techniques including diffusion tensor imaging (DTI), resting-state functional MRI (fMRI), and arterial spin labelling (ASL) imaging. Finally, we discuss new approaches for MSA research such as multimodal neuroimaging strategies and how such markers may be applied in clinical trials to provide crucial data for accurately selecting patients and to act as secondary outcome measures.

## Introduction

Multiple system atrophy (MSA) is an adult-onset, neurodegenerative disorder characterised by parkinsonism, ataxia, and dysautonomia. The neuropathological hallmark is alpha-synuclein-positive glial cytoplasmic inclusions (GCIs) with degeneration of the striatal, nigral, and olivopontine structures. The deposition of alpha-synuclein links MSA with other synucleinopathies, including idiopathic Parkinson’s disease (IPD) and dementia with Lewy bodies (DLB). However, clinically, there is more commonly overlap with atypical parkinsonian syndromes such as progressive supranuclear palsy (PSP) and corticobasal syndrome (CBS).

Diagnostic accuracy in multiple system atrophy (MSA) varies greatly between different centres from as little as 29% up to 86% [1–4], despite well-established diagnostic criteria [5]. While a definite MSA diagnosis can only be reached with post-mortem confirmation of GCIs in a well-defined pattern, a *probable* MSA diagnosis is considered when a poor levodopa-responsive parkinsonian syndrome (MSA-P) and/or a cerebellar syndrome (MSA-C) is associated with autonomic failure. A possible MSA diagnosis is defined when sporadic adult-onset parkinsonism (MSA-P), or a cerebellar syndrome (MSA-C) is accompanied by autonomic dysfunction and at least one item from a list of additional red-flag features [5, 6]. These include structural and functional neuroimaging changes that have become established over the last two decades as useful diagnostic markers. However, while such abnormalities are helpful diagnostically, brain-imaging research in MSA has expanded to examine a variety of post-processing techniques and more advanced imaging modalities that may potentially lead to much improved biomarkers for diagnosis and disease progression. In this review, we describe how different neuroimaging tools have been used to identify novel MSA biomarkers, highlighting their advantages and limitations.

## Structural MR imaging

Historically, most MSA neuroimaging research has been focused on the grey matter atrophy pattern seen on structural T1 MRI and signal changes seen on T2, FLAIR, and T2* MRI. The main aims of these studies have been to: (a) provide a diagnostic MSA atrophy pattern useful for consensus criteria; (b) distinguish between the two clinical forms MSA-P and MSA-C; and (c) improve the differential diagnosis with other neurodegenerative conditions that mimic MSA, especially with IPD, DLB, PSP, and CBS.

**Fig. 1:**
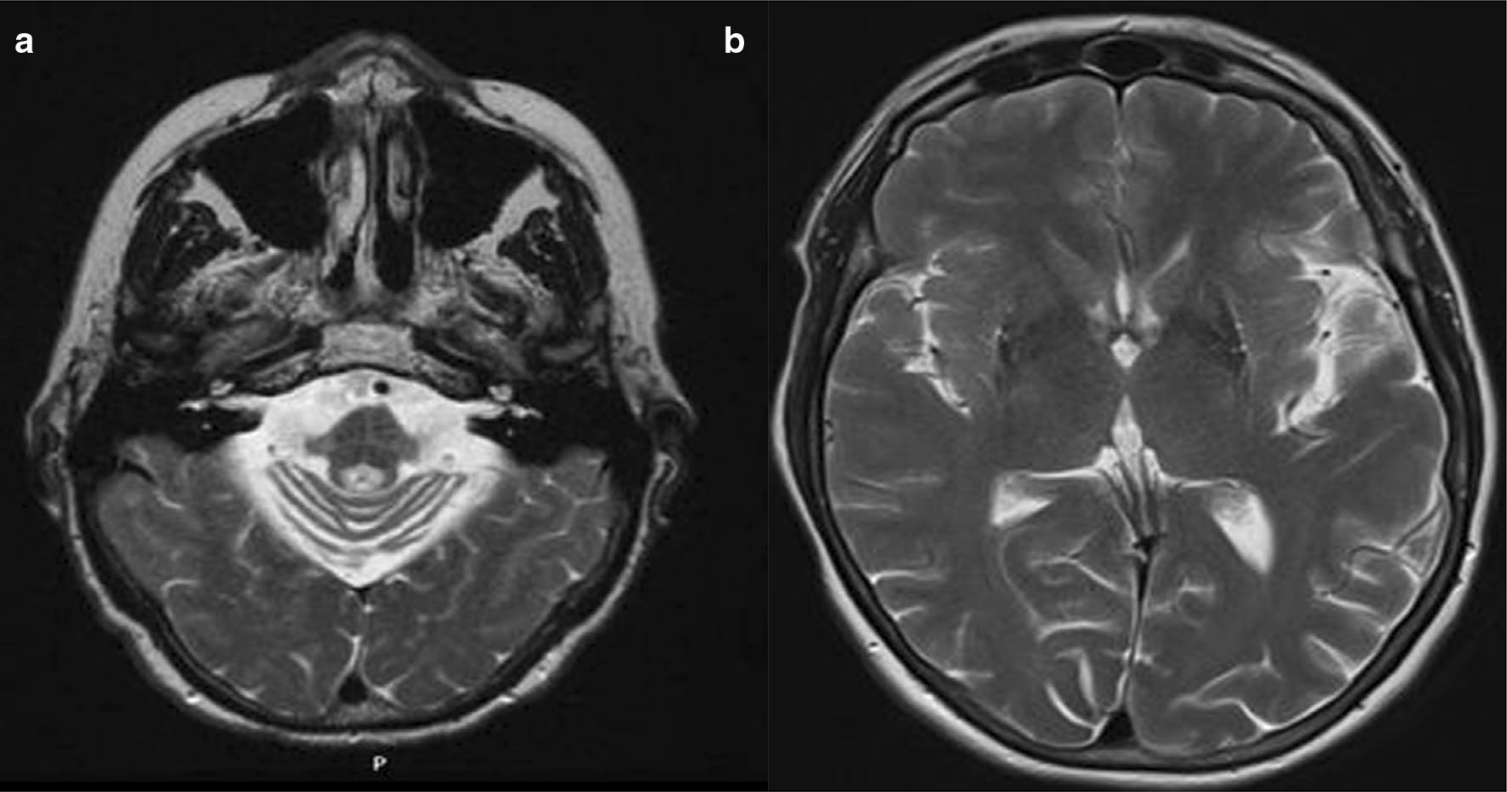
**a** ‘‘Hot cross-bun sign’’ seen on an axial T2-weighted MRI in a patient with MSA-C; **b** putaminal hypointensity with a hyperintense “putaminal rim” sign on an axial T2-weighted MRI in a patient with MSA-P. Images adapted from [9]

Visualisation of T1- and T2-weighted MRI by experienced neuroradiologists has been the cornerstone of MSA-imaging diagnosis for many years, and a number of ‘classical’ signs have been described. The ‘hot cross-bun’ sign is the most well known (Fig. 1a) and represents the degeneration of the pons and pontocerebellar fibres with the preservation of corticospinal tract. It appears as a hyperintense cross in the pons on T2-weighted imaging. Despite being a hallmark for MSA-C with high specificity (97%), its sensitivity is only 50% [7]. The ‘putaminal rim’ sign is another well-described imaging feature of MSA that has acquired its own name (Fig. 1b). The presence of a hyperintense rim to the putamen on T2*-weighted imaging is seen in MSA-P and has the highest specificity (90%) for this clinical subgroup but only 72% sensitivity [8]. It can also be a normal finding on 3T T2-weighted MRI.

Other putaminal changes described in MSA-P include atrophy (seen on T1) (sensitivity 83%, specificity 87%), and hypointensity (sensitivity 89%, specificity 70%—Fig. 1b) [8], while in MSA-C, there are changes infratentorially with hyperintensity of the middle cerebellar peduncle (MCP) and atrophy of the cerebellum and brainstem (particularly the MCP and pons) [10] (sensitivity 100%, specificity 82% for brainstem atrophy) [11]. In fact, a combination of these signs can be seen in the majority of MSA patients at different stages of the disease, independent of the initial clinical phenotype [8, 12].

While none of these signs are pathognomonic, their presence has been shown to have high specificity but lower sensitivity in differentiating MSA from other disorders [13], e.g., the presence of MCP hyperintensity has a specificity of 100% and sensitivity of 85% when compared with IPD, PSP, and normal aging controls [14].

Simply applied quantitative measures have been shown to improve diagnostic accuracy. A study that measured MCP atrophy as a reduction of the MCP width (< 8 mm in sagittal sections) showed 100% sensitivity and specificity in differentiating MSA from IPD patients [15, 16], while another study investigating the MCP/superior cerebellar peduncle (SCP) ratio had a sensitivity of 90% and specificity of 94% when comparing MSA-P to PSP [17]. Adding in measurements of the pons and midbrain allowed one study to define an MR parkinsonism index [= pontine area/midbrain area)*(MCP/SCP)] which differentiated MSA-P from PSP and IPD with high sensitivity and specificity [17].

More detailed quantitative methods of assessing cross-sectional grey matter atrophy analyses have been applied in research settings. These have usually consisted of either Region-of-Interest (ROI) volumetric analyses (performed either manually or in a more automated manner), or whole brain analyses such as voxel-based morphometry (VBM) [18].

**Fig. 2:**
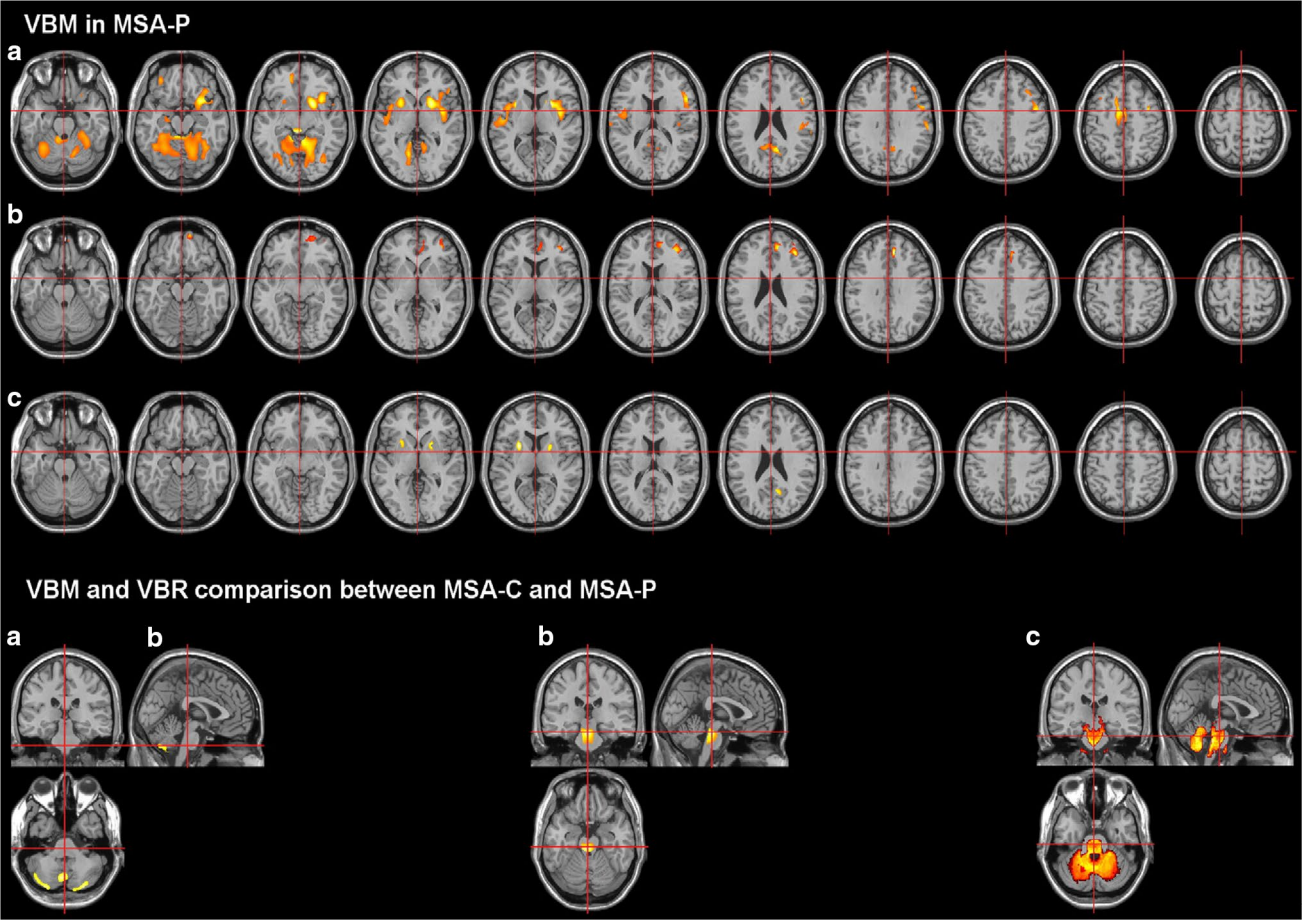
VBM in MSA-P. VBM and VBR comparison between MSA-C and MSA-P. Images are in the neuroradiological orientation (the left side of the images refers to the right side of the brain). VBM in MSA-P **a** grey matter loss, **b** correlation of grey matter loss with disease stage, and **c** increase of white matter. VBM and VBR comparison between MSA-C and MSA-P: the images display regions with more pronounced changes in MSA-C than in MSA-P. **a** Grey matter loss, **b** white matter loss, and **c** reduced relaxation rate. Images reproduced from [26]

ROI studies using semiautomatic segmentation and MRI volumetry (MRV) techniques showed a combination of supra- and infratentorial volume loss including striatum, brainstem, and cerebellum in MSA [19, 20]. A more accurate differentiation between MSA and other parkinsonian syndromes was achieved with the application of a stepwise discriminant analysis [11, 19]. Volume loss in the basal ganglia and infratentorial brain regions have been confirmed by VBM studies [21–24]. VBM is an automated method of measuring neuroanatomical changes in the grey matter using 3D volumetric T1-weighted MR imaging. Compared to controls, selective cortical atrophy involving the primary and higher order motor areas, prefrontal cortex, and insula was identified in MSA-P cases [21] (Fig. 2) and confirmed on a longitudinal VBM study [22]. A meta-analysis assessed the use of VBM in differentiating MSA-P, IPD, and normal controls [25]: although a different pattern and localization of grey matter reduction was identified in the MSA-P versus IPD group (atrophy in the putamen and claustrum), the differences were not significant in a subgroup analysis including only patients in early stages with a mean disease duration of less than three years.

**Fig. 3:**
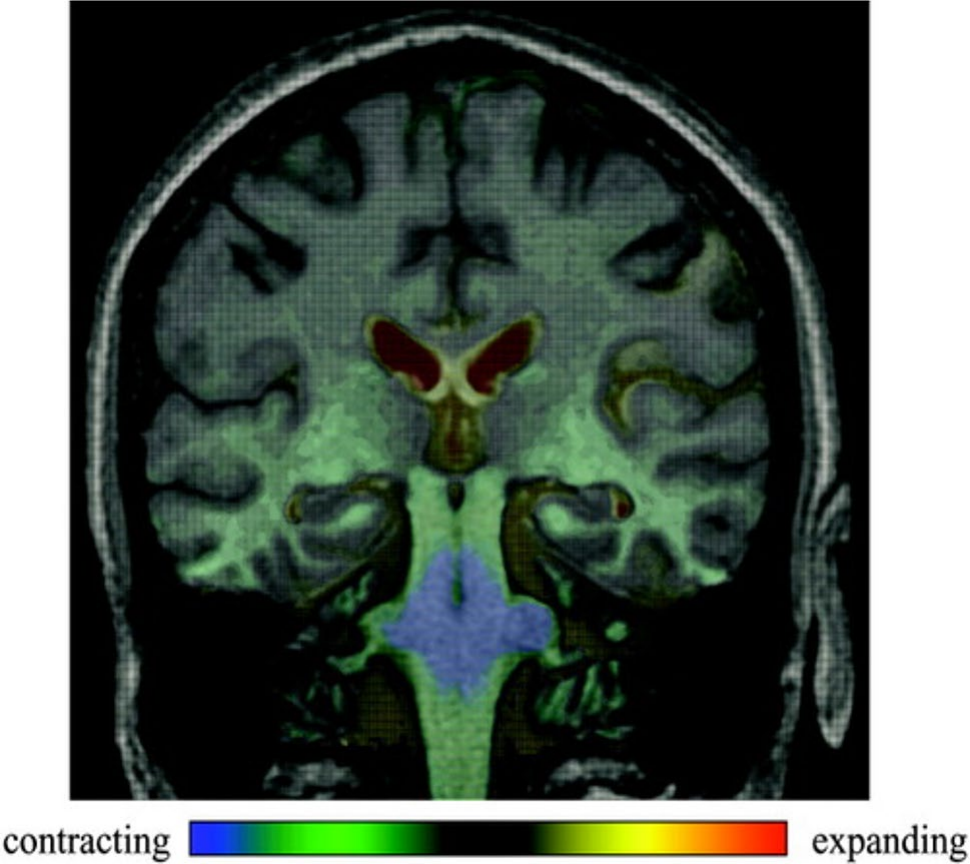
Delineating the sites and progression of in vivo atrophy in multiple system atrophy using fluid-registered MRI. Coronal MRI scan with voxel-compression-mapping overlay to demonstrate areas undergoing atrophy. Greatest rates of atrophy are demonstrated in the pons and middle cerebellar peduncles and the immediately adjacent midbrain and medulla. Increased atrophy, but at a slower rate, is seen in the upper midbrain and lower medulla. Even slower, but definitely pathological atrophy rates are seen in both temporal lobes. Ventricular enlargement is also shown. Image reproduced from [32]

Longitudinal analyses of atrophy rates are limited (Fig. 3). These studies are important, as the atrophy rate is an important quantitative marker of disease progression that has been successfully implemented in other neurodegenerative conditions as an outcome measure in clinical trials [27–29]. Only two studies have assessed whole brain atrophy rate (WBAR) in MSA [30, 31]. Although these had a short follow-up and assessed a small number of cases, they showed that the WBAR was higher from the early stages of MSA (and PSP) compared with IPD, suggesting that this could be used as an unbiased outcome measure for monitoring the disease course in future clinical trials. In addition, as MSA is a rapidly progressive disorder, using imaging measurements improves reliability compared to clinical disease rating scores [11, 19, 30].

## Measures of signal change

### DWI

**Fig. 4:**
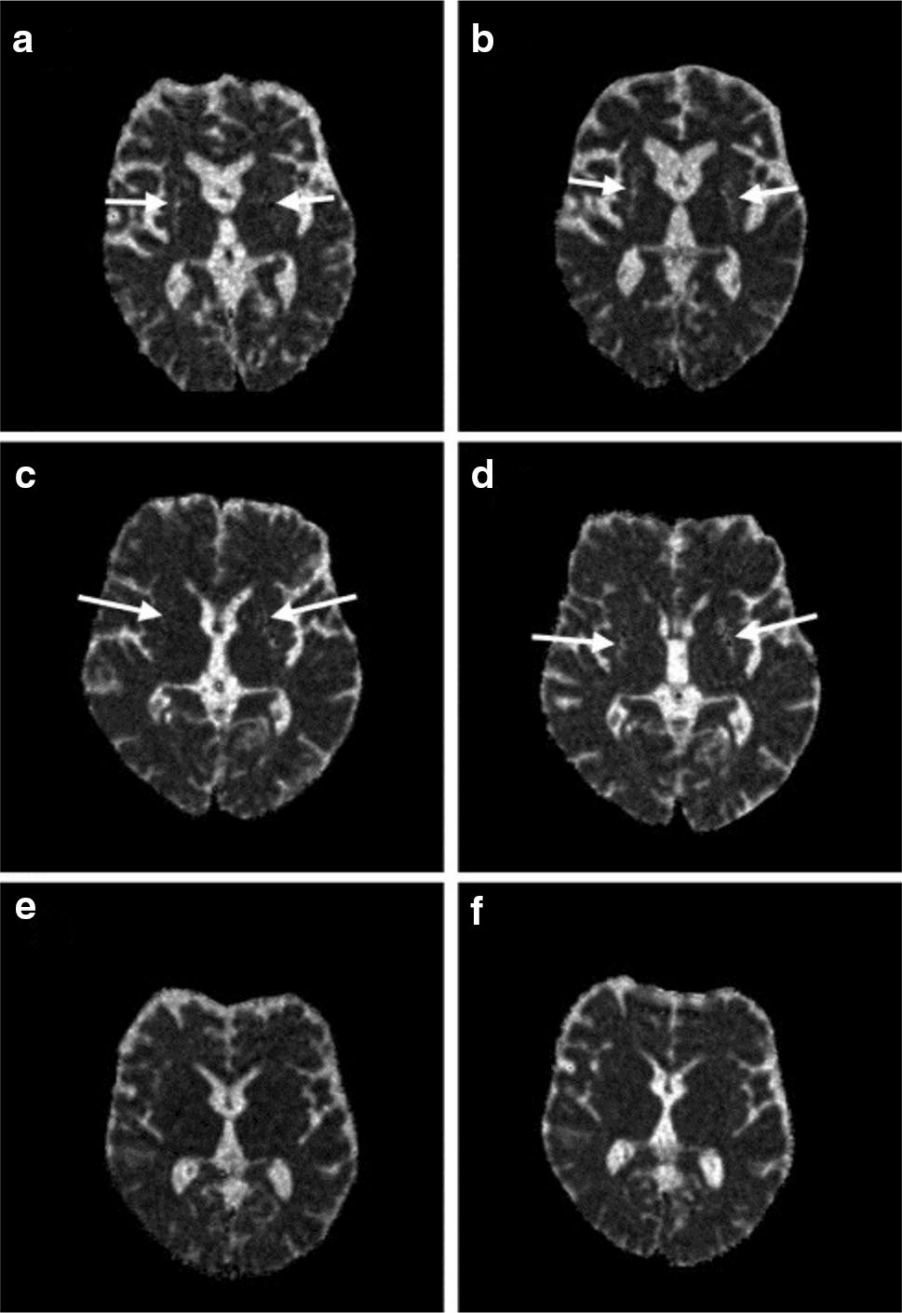
Progression of putaminal degeneration in MSA using diffusion MR. Trace (*D*) maps at the level of mid-striatum in individual patients with the Parkinson variant of multiple system atrophy (MSA-P) (*n* = 2; **a** baseline; **b** follow-up in one patient; **c** baseline; **d** follow-up in another patient) and Parkinson’s disease (PD) (**e** baseline; **f** follow-up). Note the diffuse hyperintensity—corresponding to increased Trace (*D*) values—in the putamina of the patient with MSA-P (arrows in **a**–**d**) which are increased at follow-up (**b, d**) compared to baseline examination (**a, c**). The PD patient shows no increased Trace (*D*) values in the putamen, neither at baseline (**e**) nor at follow-up (**f**). Images reproduced from [39]

Diffusion-weighted imaging (DWI) uses water molecule movement and calculates the apparent diffusion coefficient (ADC) in tissue as a measure of integrity. In neurodegeneration and ischemia, the random movement of water molecules is increased. One of the most promising DWI markers for MSA is raised putaminal diffusivity in MSA-P compared to PD, even in early stages of disease [33–36] (Fig. 4). DWI was also helpful in differentiating MSA-P from PSP where increased regional ADC in the MCP and pons in MSA-P, compared to PSP, had 91% sensitivity and 84% specificity [37]. DWI-measured progression of striatal and extrastriatal degeneration including the putamen, pons, and cerebellar white matter in MSA in a longitudinal study correlated well with disease duration and severity at 1-year follow-up [38].

### MTR

Magnetization transfer imaging allows brain structure segmentation and ratio (MTR) calculation of a specific ROI. A significant decrease in MTR of the globus pallidus, putamen, and substantia nigra has been reported in MSA compared to IPD [40]. Changes in the basal ganglia were reported in MSA-P in studies using MTR [40, 41]. Furthermore, using basal ganglia and substantia nigra changes in stepwise MRT analysis provided a good discrimination rate between PD cases and controls from the MSA and PSP group. However, the classification of individual MSA and PSP cases into disease groups was not optimal [40].

### SWI

Susceptibility-weighted imaging (SWI) is a gradient echo image that provides information about any tissue that has a different magnetic susceptibility compared to its surrounding structures such as deoxygenated blood, hemosiderin, ferritin, and calcium [42]. Compared with a standard T2* sequence, there is increased sensitivity in detecting local changes in iron content [43]. This is important in MSA, as several histopathological studies have revealed increased iron and ferritin levels in the putamen (particularly posteriorly), striatum and substantia nigra [44], and at a significantly higher amount than in IPD [45]. SWI studies have shown a much higher iron deposition in the putamen and pallidum of MSA-P compared to IPD and PSP [46, 47]. One study splitting the putamen into four regions suggested that the lower inner part is the best marker to differentiate between MSA-P and IPD [48]. Another study showed increasing iron accumulation in the putamen from posterolateral areas in the early stages to more anteromedial areas later [49]. Studies of the caudate are less clear: one study found an increased deposition of iron in the caudate nucleus of MSA-P compared to IPD [48], while a second study could not replicate these findings based on SWI alone [49]. These contradictory results may well represent differences in disease duration in the different sample groups.

### VBR

Voxel-based relaxometry (VBR) is a morphometric method that analyses the relaxation rate R2 (defined as 1/T2) derived from multi-echo T2-weighted images on a voxel by-voxel basis using the exponential relationship between the actual transverse magnetization and the relaxation rate R2. In principle, decreased R2 indicates increased water content and, therefore, provides a measure of tissue atrophy [26]. VBR analysis in MSA-C patients revealed a reduced relaxation rate R2 particularly within the cerebellum, middle cerebellar peduncles, and pons [19, 24, 50, 51].

### QSM

Quantitative susceptibility mapping (QSM) detects local susceptibility changes to metals such as iron. Studies suggested that QSM is a better tool for measuring iron levels in the tissue [52, 53]. Both the R2 and QSM are increased in MSA and PSP compared to PD and controls in several brain structures including the basal ganglia and cerebellum. However, compared to PSP, the MSA subgroup had different iron deposition patterns in the SN, thalamus, and red nucleus [54, 55]. A post-mortem study assessed the QSM and R2 in path confirmed parkinsonian disorders showing that in vivo increased R2 was significantly associated with alpha-synuclein and QSM correlated significantly with Perl’s stain for iron. However, neither measurement correlated with tau nor glial cell counts [53].

## Structural and functional connectivity

Diffusion tensor imaging (DTI) uses the motion of water to quantify changes in the microstructure of white matter tracts [56]. DTI metrics commonly include fractional anisotropy (FA), axial diffusivity (AD), radial diffusivity (RD), and mean diffusivity (MD). FA is a marker of fibre structural integrity, being reduced when neuronal fibres are destroyed causing water diffusion to occur in all directions (become isotropic). Diffusivity measures are higher [57], with AD considered a reflection of axonal loss and RD of myelin damage [58].

**Fig. 5:**
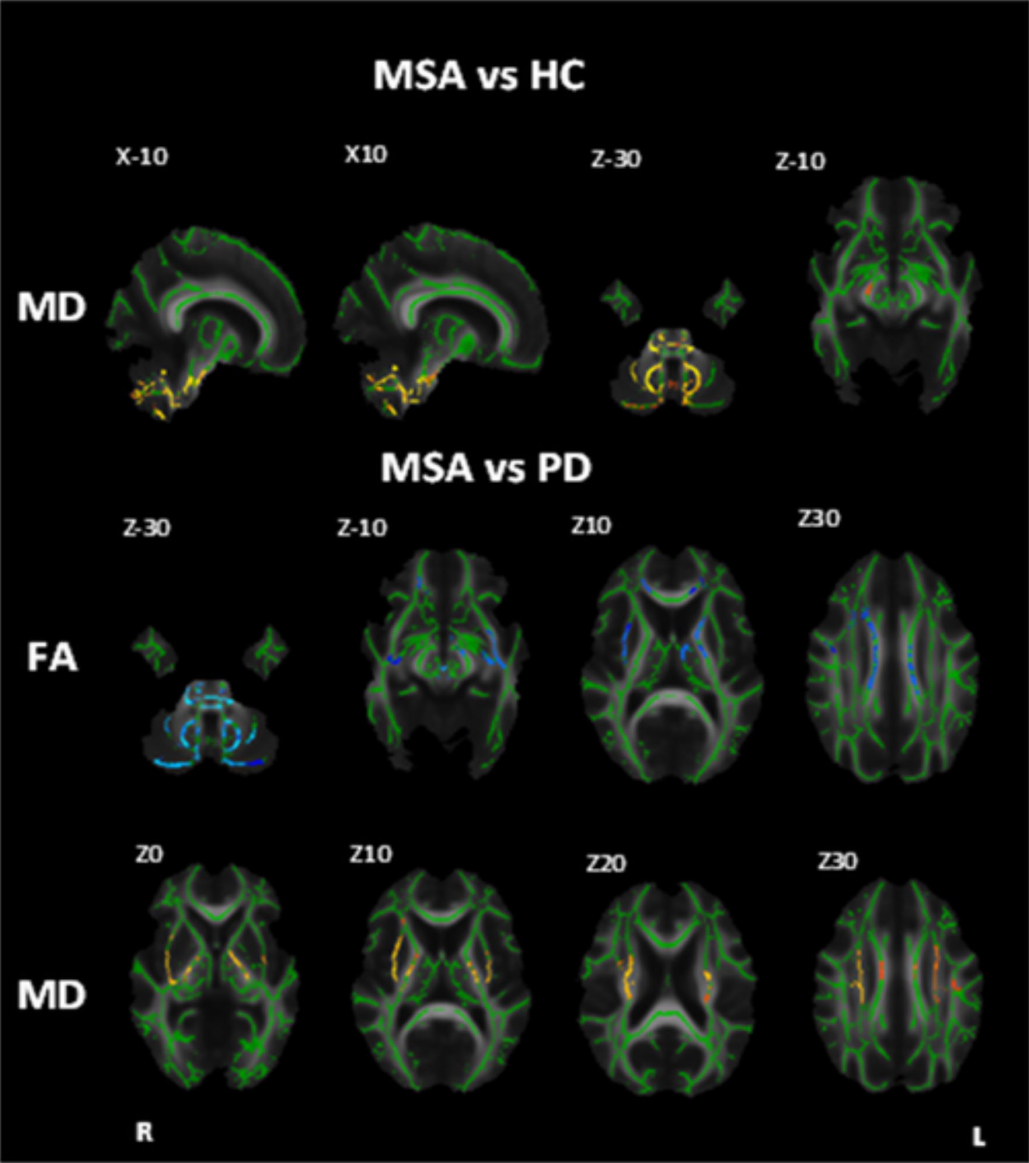
Diffusion tensor imaging in MSA. White matter maps showing regions of significant decreased fractional anisotropy and increased mean diffusivity in MSA patients when compared to healthy controls and PD (Bonferroni corrected alpha = 0.0167). Background image corresponds to the mean fractional anisotropy image of all subjects in the standard MNI152 space (radiological view). Fractional anisotropy white matter skeleton is represented by green voxels. Blue voxels represent regions of decreased FA, and yellow voxels represent regions of increased MD in the PSP group. Images reproduced from [61]

Several studies have used DTI to assess white matter tract changes in MSA. DTI shows that the cerebellum, in particular, MCP regions, and globus pallidum of MSA patients have reduced FA associated with higher MD values, compared to IPD [59, 60] (Fig. 5).

One study compared DTI in different MSA subtypes using the Diffusion Trace (*D*) generating brain maps from the MD images in the three orthogonal directions. Trace (*D*) values measured in the entire and anterior putamen were significantly higher in MSA-P than in MSA-C cases, whereas Trace (*D*) values in the cerebellum and middle cerebellar peduncle (MCP) were significantly higher in MSA-C than in MSA-P patients and controls. Furthermore, the increase of disease duration significantly correlated with increased Trace (*D*) values in the pons of MSA-P patients and in cerebellum and MCP of MSA-C patients. Both Unified Multiple System Atrophy Rating Scale (UMSARS) and Unified Parkinson’s Disease Rating Scale (UPDRS) motor scores positively correlated with entire and posterior putaminal Trace (*D*) values in MSA-P patients [35].

The hot cross-bun sign implies that the corticospinal tract (CST) remains intact in the presence of degeneration of pons and pontocerebellar fibres. However, this contradicts pathological reports that support the involvement of white matter CST in MSA. Using DTI CST, white matter changes have been shown to be present, with a marked decrease in FA and increase in MD observed in the transverse pontocerebellar fibres, the corticospinal tracts, pons, and the cerebellum. These observations correlate well with neuropathological studies [62], suggesting that DTI is much better at detecting white matter structural changes than the standard MRI sequences.

Resting-state functional MRI (rsfMRI) is a relatively new tool that, unlike task-based fMRI, assesses brain connectivity, while the subject is at ‘rest’ (lying quietly in the scanner). This technique allows the assessment of ‘functional’ connectivity and networks by visualising synchronised neuronal activation (based on the blood oxygenation level or BOLD signal) between spatially distinct brain regions [63]. The most well-defined network is the default mode network [64], although multiple other ‘intrinsic connectivity networks’ have been described including sensorimotor, visual, language, attention, and salience networks [65–70]. There are currently only a small number of studies using rsfMRI in MSA. The first such study showed that the networks most affected in MSA are the default mode and sensorimotor networks [71]. 20 clinically probable MSA patients were analysed alongside 9 healthy controls, using a regional homogeneity (ReHo) method to investigate neuronal networks in resting state. ReHo changes have been described in IPD, including sensorimotor networks [72], but in the IPD group, ReHo was reduced in medial PFC and SMA, compared with an increase in the same areas in MSA. Although these are very small studies and require further replication, they could be significant. However, other pathways affected in MSA, e.g., the olivopontocerebellar and the nigrostriatal networks, were not assessed, so much remains to be explored.

Whole brain connectivity analysis was also used to monitor response to repetitive transcranial magnetic stimulation (TMS) in MSA patients in a small study [73]. Patients were randomised to 10 sessions of TMS targeting the motor cortex area or sham TMS. Patients receiving active TMS showed changes in several networks including the default mode, cerebellar, and limbic networks. Interestingly, the positive changes in the functional networks were associated with improved motor symptoms in the TMS-treated group [73].

## Measures of perfusion

Arterial spin labelling (ASL) is a new MRI technique that uses magnetically labelled water molecules in the blood to trace cerebral blood flow (CBF). As CBF is directly linked to metabolic activity, ASL is a good non-invasive, radiation-free, low-cost marker of perfusion. Whole brain CBF maps can be calculated from the acquired data allowing group comparisons [74]. In PD, ASL has been shown to be an alternative to PET and SPECT for assessing perfusion [75, 76]. ASL has not yet been applied to MSA, but recent experience in other neurodegenerative disorders, including various parkinsonian syndromes, is encouraging, suggesting that ASL may prove a useful and safe neuroimaging tool in the future.

## Multimodal imaging and the future

Complex molecular processes such as neurodegeneration require comprehensive neuroimaging protocols to accurately describe and track disease progression and studies are now starting to combine multiple imaging modalities. Combining T2* relaxation rates with DTI metrics has revealed significant changes in the putamen of MSA compared with IPD. Comparing the clinical subtypes, the MSA-P group showed a higher MD in the putamen compared to IPD and MSA-C. Importantly, the combination of the two methods assessing structural integrity (T2* and MD) provided 96% accuracy in differentiating IPD cases from MSA-P [33]. In a group of early stage atypical parkinsonian syndrome patients with unknown diagnosis, a prospective study using both conventional T1/T2-MRI (regions of atrophy and signal changes) and DTI (MD and FA metrics) measures assessed the diagnostic accuracy when both techniques were used. A diagnosis was reached after clinical follow-up. As with the previous study, significantly higher MD in the putamen was present in the MSA-P compared to the rest of the group, and diagnostic accuracy increased when the DTI metrics were added [77]. Similarly, adding SWI data to conventional structural MRI improved the accuracy of identifying the MSA cases from the atypical parkinsonism group [48].

Although not yet applied to MSA, a combination of MRI, DTI, fMRI, and ASL has improved diagnostic accuracy in other neurodegenerative disorders, e.g., AD and FTD based on structural and functional white matter involvement [78]. A similar approach could provide significant qualitative and quantitative markers of disease and progression for MSA, where the white matter tracts are affected early in the disease course.

## Conclusion

In recent years, there has been significant progress in neuroimaging techniques and their application to research into neurodegenerative disorders. In MSA, most studies in the past have focused on the volumetric and structural patterns of the disease. However, improved diagnostic accuracy and novel disease progression markers have been reported using new connectivity and functional techniques. None of these tools alone are able to provide all the necessary quantitative and qualitative measured outcomes. However, a multimodal approach using these innovative technologies as part of the diagnostic toolkit seems likely to offer the best path for future progress in both clinical diagnosis and research into MSA.
